# RegPrecise 3.0 – A resource for genome-scale exploration of transcriptional regulation in bacteria

**DOI:** 10.1186/1471-2164-14-745

**Published:** 2013-11-01

**Authors:** Pavel S Novichkov, Alexey E Kazakov, Dmitry A Ravcheev, Semen A Leyn, Galina Y Kovaleva, Roman A Sutormin, Marat D Kazanov, William Riehl, Adam P Arkin, Inna Dubchak, Dmitry A Rodionov

**Affiliations:** 1 Lawrence Berkeley National Laboratory, Berkeley 94710, CA, USA; 2 A.A. Kharkevich Institute for Information Transmission Problems, Russian Academy of Sciences, Moscow 127994, Russia; 3 Sanford-Burnham Medical Research Institute, La Jolla 92037, CA, USA; 4 Department of Bioengineering and Bioinformatics, Lomonosov Moscow State University, Leninskye Gory 1-73, Moscow 119992, Russia

**Keywords:** Regulatory network, Regulon, Transcription factor, Riboswitch, Comparative genomics, Bacteria

## Abstract

**Background:**

Genome-scale prediction of gene regulation and reconstruction of transcriptional regulatory networks in prokaryotes is one of the critical tasks of modern genomics. Bacteria from different taxonomic groups, whose lifestyles and natural environments are substantially different, possess highly diverged transcriptional regulatory networks. The comparative genomics approaches are useful for *in silico* reconstruction of bacterial regulons and networks operated by both transcription factors (TFs) and RNA regulatory elements (riboswitches).

**Description:**

RegPrecise (http://regprecise.lbl.gov) is a web resource for collection, visualization and analysis of transcriptional regulons reconstructed by comparative genomics. We significantly expanded a reference collection of manually curated regulons we introduced earlier. RegPrecise 3.0 provides access to inferred regulatory interactions organized by phylogenetic, structural and functional properties. Taxonomy-specific collections include 781 TF regulogs inferred in more than 160 genomes representing 14 taxonomic groups of Bacteria. TF-specific collections include regulogs for a selected subset of 40 TFs reconstructed across more than 30 taxonomic lineages. Novel collections of regulons operated by RNA regulatory elements (riboswitches) include near 400 regulogs inferred in 24 bacterial lineages. RegPrecise 3.0 provides four classifications of the reference regulons implemented as controlled vocabularies: 55 TF protein families; 43 RNA motif families; ~150 biological processes or metabolic pathways; and ~200 effectors or environmental signals. Genome-wide visualization of regulatory networks and metabolic pathways covered by the reference regulons are available for all studied genomes. A separate section of RegPrecise 3.0 contains draft regulatory networks in 640 genomes obtained by an conservative propagation of the reference regulons to closely related genomes.

**Conclusions:**

RegPrecise 3.0 gives access to the transcriptional regulons reconstructed in bacterial genomes. Analytical capabilities include exploration of: regulon content, structure and function; TF binding site motifs; conservation and variations in genome-wide regulatory networks across all taxonomic groups of Bacteria. RegPrecise 3.0 was selected as a core resource on transcriptional regulation of the Department of Energy Systems Biology Knowledgebase, an emerging software and data environment designed to enable researchers to collaboratively generate, test and share new hypotheses about gene and protein functions, perform large-scale analyses, and model interactions in microbes, plants, and their communities.

## Background

Fine-tuned regulation of gene transcription in response to extracellular and intracellular signals is a key mechanism for successful adaptation of microorganisms to changing environmental conditions. Activation and repression of gene expression in bacteria is usually mediated by DNA-binding transcription factors (TFs) that specifically recognize TF-binding sites (TFBSs) in upstream regions of target genes, and also by various regulatory RNA structures including *cis*-acting metabolite-sensing riboswitches and attenuators encoded in the leader regions of target genes. Genes and operons directly co-regulated by the same TF (or by RNA motifs from the same structural family) form a so called regulon [1]. All regulons together operated in the same genome form a transcriptional regulatory network (TRN) of a cell.

Computational methods based on the comparison of TFBSs in related species proved to be efficient for predicting transcriptional regulons in Bacteria [2-5]. To address the challenge of regulatory network reconstruction in ever growing number of sequenced microbial genomes, we recently developed a strategy for fast and accurate comparative reconstruction of large-scale TRNs and implemented it in the RegPredict web server [6]. First, the bacterial species tree is subdivided into small taxonomic groups, and a subset of 5–15 representative genomes from each group is selected. Second, semi-automatic reconstruction of reference regulogs (orthologous regulons) in these selected genomes is carried out using both known TF-binding motifs and ab initio predicted novel DNA motifs (reviewed in [1]). Resulting regulons are characterized by a TF, predicted DNA-binding motif, and a set of target genes/operons together with associated TFBSs in their upstream regions. A regulog, that is a group of regulons operated by the orthologous TFs in closely related genomes, represents the main outcome of the RegPredict-based analysis. The reference regulogs are then used for an automatic propagation of the captured regulatory interactions into new genomes from the same taxonomic group.

By applying this computational approach to a growing number of complete bacterial genomes, we inferred high-quality genome-scale TRNs for diverse taxonomic groups of bacteria, namely *Shewanella*, *Thermotoga*, *Desulfovibrio*, *Bacillus*, *Lactobacillus*, *Streptococcus* and *Staphylococcus* spp. [7-20]. To provide public access to the collections of transcriptional regulons reconstructed via the RegPredict web server, some time ago we had developed the first version of the RegPrecise database for capturing and visualization of the curated regulon inferences [21]. Recently added RegPrecise web services [22] allow for programmatic access to the transcriptional regulatory data.

Here we present RegPrecise Version 3.0 with significantly increased biological data content and novel database features. The current database contains more than 1500 regulogs including ~400 regulogs controlled by RNA regulatory motifs in 24 taxonomic groups, and ~800 TF-operated regulogs in 14 taxonomic collections. Novel features of RegPrecise 3.0 include controlled vocabularies for effectors and metabolic pathways, detailed classifications for TF proteins and RNA motif families, and several types of visualization for genome-wide regulatory networks. RegPrecise 3.0 is a largest and fast growing web resource for comparative genomics of transcriptional regulation in Bacteria. It is highly valuable both for experimental biologists studying mechanisms of transcriptional regulation in bacteria, and computational biologists interested in modeling metabolic and regulatory networks.

## Construction and content

The RegPrecise database contains detailed information on regulatory interactions and transcriptional regulons inferred by a comparative genomics in diverse bacterial genomes [21]. In addition to TF-operated regulons, the updated version of the database includes the inferred regulons for RNA regulatory motifs (riboswitches) [23]. Below we describe the database structure and data organization, and present new features and statistics for significantly updated RegPrecise 3.0 content.

**Figure 1:**
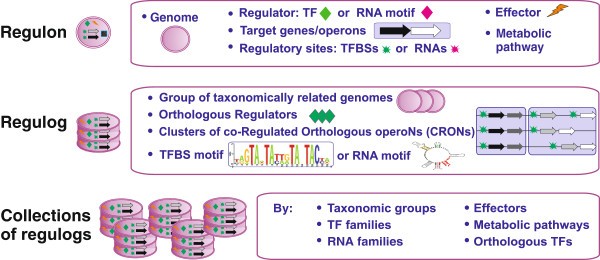
Data organization in RegPrecise.

The database has the following hierarchical data organization: (i) a regulon; (ii) a regulog; and (iii) a collection of regulogs (Figure 1). A regulon is a basic unit of the database that represents a set of target genes/operons that are co-regulated by the same regulator (TF or RNA motif) in a particular genome. The description of each regulon in RegPrecise also includes an alignment of TF binding sites (or RNA regulatory sites). A regulog represents a set of regulons under control of orthologous regulators in a group of taxonomically related genomes. Each TF-operated regulog has a TFBS motif represented as a sequence logo or an alignment.

**Figure 2:**
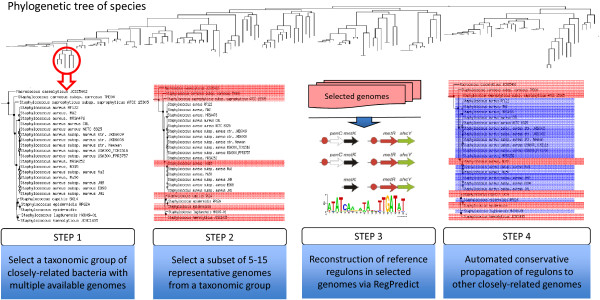
Comparative genomics-driven strategy for regulon inference in RegPrecise.

Our strategy for regulon reconstruction in RegPrecise includes four steps (Figure 2): (i) selection of a group of closely-related bacteria on the species tree; (ii) selection of a subset of diverse genomes that represent a given taxonomic group; (iii) reconstruction and manual curation of reference regulogs in the selected genomes; (iv) accurate automatic propagation of the reference regulogs to the large set of closely-related genomes from the same taxonomic group. Accordingly, the RegPrecise 3.0 database includes two major sections: (1) *reference regulog collections*, and (2) *propagated regulons.* Below we describe data construction and content for each of these sections.

### Building reference regulog collections

We use RegPredict web server [6] for careful comparative analysis and manual curation of each regulog in RegPrecise. RegPredict allows for the simultaneous analysis of multiple microbial genomes and integrates information on gene orthologs, operon predictions, and functional gene annotations. It implements two well-established workflows for inference of TF-operated regulons: i) regulon reconstruction for known TFBS motifs, and ii) ab initio inference of novel TFBS motifs and regulons. For experimentally characterized regulons, we used training sets of known TFBSs collected from literature and other regulatory databases [24-27] to build a position weight matrix (PWM) for a TFBS motif. Novel TFBS motifs were identified by Discover profile tool of RegPredict using sets of potentially co-regulated genes. Constructed PWMs for DNA motifs (both known and ab initio predicted) were used to scan each selected genome and identify genes with candidate regulatory sites in upstream regions. Each predicted regulatory interaction was analyzed for conservation across the group of closely related genomes using the Clusters of co-Regulated Orthologous operoNs (CRONs) in RegPredict. For each analyzed regulon, the set of constructed CRONs was prioritized based on the level of conservation of regulatory interactions, emphasizing the most prominent regulon members. At the next step, we conducted the functional and genomic context analysis of each CRON using the advanced web interface facilitating the decision on CRON inclusion in the final regulog model. Combining all accepted CRONs for a given TFBS motif produces the reconstructed TF regulog for a group of target genomes.

We utilized a similar workflow for reconstruction of regulogs operated by RNA motifs. First, RNA regulatory sites were identified in the studied genomes using the probabilistic covariance models for 43 RNA families from the Rfam database [28] and the Infernal program [29]. Then, the identified candidate RNA sites were uploaded into RegPredict and used for regulog reconstruction using the similar CRON-based approach as for TF regulogs. Thus, each inferred RNA regulog includes all genes/operons that are preceded by a candidate Rfam-family motif in a studied taxonomic group of genomes [23].

### Collections of regulogs

All reference regulogs are classified into *collections* of six biological types briefly described below.

*Taxonomic collections* represent results of large-scale reconstructions of both TF- and RNA-operated regulogs in narrow taxonomic groups of bacteria. RegPrecise 3.0 contains 14 taxonomic collections covering major phyla of Bacteria and including 781 regulogs (Table 1). These data represent a major expansion since the 1.0 version that had only two taxonomic collections [21]. The reconstructed genome-wide TRNs for bacteria from six taxonomic collections (*Shewanella*, *Staphylococcus*, *Bacillales*, *Streptococcaceae*, *Lactobacillaceae* and *Thermotogales*) have been described in our research papers [10,12,13,19,20], whereas publications on other taxonomic collections are currently in preparation. The reconstructed genome-specific TRNs utilize and expand experimental knowledge on regulatory interactions accumulated in the RegTransBase database developed in our group [25], and other specialized databases (DBTBS [27], RegulonDB [26], CoryneRegNet [24]). For instance, the *Bacillus subtilis* TRN was expanded by ~300 new target genes and 36 novel TF regulons that await future experimental validation [10]. The genome-wide TRNs from taxonomy collections are useful for building predictive metabolic models with regulatory constraints.

**Table 1:** Taxonomic collections of curated genome-wide TRNs in diverse microbes

**Taxonomic group**	**Phylum**	**Reference genomes**	**TF regulogs**	**RNA regulogs**	**TF binding sites**	**RNA sites**	**Regulated genes**^**1**^	**Genes per genome**^**2**^
**Bacillales**	Firmicutes	11	134	39	3815	668	7301	664
**Staphylococcus**	Firmicutes	7	48	29	1965	288	3329	476
**Lactobacillaceae**	Firmicutes	15	79	39	1811	581	3784	252
**Streptococcaceae**	Firmicutes	15	69	29	3118	400	5652	377
**Clostridiaceae**	Firmicutes	20	7	40	303	968	2489	124
**Enterobacteriales**	Proteobacteria	12	87	18	7365	188	9028	752
**Shewanella**	Proteobacteria	16	80	15	8450	291	10817	676
**Ralstonia**	Proteobacteria	6	24	10	574	66	1297	216
**Desulfovibrionales**	Proteobacteria	10	92	9	1942	72	3368	337
**Thermotogales**	Thermotogae	11	33	13	642	88	2153	196
**Corynebacteriaceae**	Actinobacteria	8	45	13	937	80	1624	203
**Bacteroidaceae**	Bacteroidae	11	35	2	667	84	1797	163
**Chloroflexi**	Chloroflexi	5	30	17	314	98	1014	203
**Cyanobacteria**	Cyanobacteria	14	18	11	1032	86	1442	103
**Total:**	**-**	**161**	**781**	**284**	**32935**	**3958**	**55095**	**342**

^1^Total number of regulated genes in all TF- and RNA operated regulons.

^2^Average numbers of regulated genes per genome were calculated for each studied taxonomic group; the average numbers for all lineages are given in the last line.

*TF collections* contain regulogs for a selected subset of TFs conserved in more than three taxonomic groups. Each TF collection represents all reconstructed regulogs for a given set of orthologous TFs across different taxonomic groups of bacteria. The RegPrecise 3.0 contains 40 TF collections (an increase of 31 TFs since the previous database version) that include both widespread TFs such as NrdR, LexA and Zur present in more than 25 diverse taxonomic groups, and narrowly distributed TFs such as Irr in α-proteobacteria and PurR in γ-proteobacteria. Altogether, the orthologous TF collections include 443 regulogs that are valuable for comparative and evolutionary analysis of TF binding motifs and regulon contents, as illustrated by our previous publications on comparative genomics analyses of numerous TFs including HexR [11], Rex [14], NrdR [17], NrtR [30], NiaR [31], KdgR and ExuR [32], AraR and XylR [33], PsrA and LiuR [7], NsrR and NorR [16], Irr and IscR [18], BirA [34], and PaaR [35].

*TF family collections* provide structural classification of more than 7000 TFs that belong to more than 1000 reconstructed TF regulogs. All studied TFs were classified into 55 protein families based on their domain composition in the Pfam [36], COG [37] and Superfamily [38] libraries (see TF families classification and domains architecture in Additional file 1). Annotations of TF protein domains were collected from the MicrobesOnline database [39]. Each TF family was characterized by at least one DNA-binding domain and one or several additional domains involved in effector sensing and/or dimerization. In RegPrecise 3.0, we provide a short summary with literature citations for each of the 55 TF families. The TF family collections covers both large and diverse families such as LacI, GntR, and TetR that contain more than 100 TF regulogs, and narrow families such as ArgR, BirA and LexA containing orthologous TFs of the same function. These collections are valuable for evolutionary analysis of TF binding site motifs and effector specificities within the same TF family.

*RNA family collections* is a novel section that provides an access to near 400 reconstructed regulogs operated by the RNA regulatory elements in more than 250 bacterial genomes from 24 taxonomic groups. The RegPrecise 3.0 includes RNA family collections for 43 Rfam families. For each collection we provide a short biological summary with literature citations and cross references to the Rfam database [28]. Among the analyzed regulatory RNAs are 15 metabolite-sensing riboswitches, 6 ribosomal operon leaders, 4 amino acid-responsive attenuators, and multiple *cis*-acting regulatory RNAs of yet unknown regulatory mechanisms. The large collection of T-box regulogs for amino acid metabolism was additionally classified by amino acid specificities of T-boxes deduced from their multiple sequence alignment. The detailed evolutionary analysis of regulog content from the RNA family collections was recently published [23].

*Effector and Pathway collections* represent two novel functional classifications of regulogs in RegPrecise 3.0. We used controlled vocabularies of 255 regulatory effectors and 235 metabolic pathways to organize collections of these two types. Effectors were retrieved from manually curated annotations of TF- and RNA-operated regulogs in RegPrecise, and assigned to 12 higher-level categories. These categories include amino acids, aminoacyl-tRNAs, antibiotics, carbohydrates, coenzymes, heterocyclic compounds, inorganic chemicals, lipids and fatty acids, nucleotides and nucleosides, organic chemicals, peptides and proteins and other factors (according to MeSH headings). Metabolic pathways and biological processes were assigned to regulogs based on the analysis of functional regulon content and experimental data from the literature. The RegPrecise 3.0 contains 235 pathways classified into 23 functional categories according to the biological subsystems classification from the SEED database [40]. Two largest functional categories of regulogs in RegPrecise are those involved in the metabolism of carbohydrates and amino acids.

### Propagated TF regulons

The obtained reference TF regulogs were used for large-scale annotation of regulatory interactions in closely related genomes by using an automated conservative propagation procedure. For each taxonomic collection including manually curated regulons in the selected subset of genomes, we selected an expanded set of genomes from the same taxonomic lineage that are available in the MicrobesOnline database [39]. To propagate a particular TF regulog to a target genome, we identified orthologs for both a TF gene and each of the previously described members of a reference TF regulog using the pre-computed ortholog groups in MicrobesOnline. For the identified gene orthologs in target genomes, we perform search for candidate TFBSs in their upstream regions (from −350 to +50 bp relative to the start codon, excluding the coding regions of upstream genes). For search of putative binding sites we utilized a PWM that is associated with the reference regulog and was used for its original reconstruction. Each propagated regulon has one or more candidate regulated genes, their upstream binding sites, and, in most cases, an attributed orthologous TF. Moreover, we explicitly provide comparative genomics evidences supporting for each predicted regulatory interaction. Possible operon structures of the identified regulated genes have not been studied, thus the propagated regulons are still preliminary and need to be improved by operon prediction in the future. Nevertheless, the regulon propagation procedure is considered to be accurate and conservative, since it relies on the manually curated regulons and does not make an attempt for automatic prediction of new members of regulon.

As result, the conservative propagation procedure was applied to 640 genomes from 14 taxonomic groups with available genome-wide collections of reference TF regulogs (Table 2). Three largest taxonomic groups with propagated TF regulons were Enterobacteriales (160 genomes), Bacillales (68 genomes) and Streptococcaceae (69 genomes). The *propagated regulons* section in RegPrecise 3.0 represents a large set of draft TF regulons annotated in all available genomes within the analyzed taxonomic groups of bacteria (Table 2).

**Table 2:** Collections of TF regulons reconstructed by conservative propagation

**Propagated regulons**			**Reference regulons**			
**Taxonomic group**	**Genomes**	**TF regulons**	**Regulog collection**	**Genomes**	**TF regulogs**	**TF regulons**
**Bacillales**	68	3784	**Bacillales**	11	134	844
**Staphylococcaceae**	25	876	**Staphylococcus**	7	48	271
**Lactobacillaceae**	29	873	**Lactobacillaceae**	15	79	483
**Streptococcaceae**	69	2644	**Streptococcaceae**	15	69	593
**Clostridia**	61	144	**Clostridiaceae**	20	7	51
**Enterobacteriales**	160	7735	**Enterobacteriales**	12	87	698
**Alteromonadales**	39	1444	**Shewanella**	16	80	862
**Burkholderiaceae**	74	1022	**Ralstonia**	6	24	122
**Desulfovibrionales**	11	349	**Desulfovibrionales**	10	92	392
**Thermotogales**	11	223	**Thermotogales**	11	33	239
**Corynebacteriaceae**	9	237	**Corynebacteriaceae**	8	45	209
**Bacteroidaceae**	22	254	**Bacteroidaceae**	11	35	215
**Chloroflexi**	14	139	**Chloroflexi**	5	30	107
**Cyanobacteria**	48	510	**Cyanobacteria**	14	18	180
**Total:**	**640**	**20234**	**Total:**	**161**	**781**	**5266**

## Utility and discussion

The RegPrecise 3.0 interface provides several different ways to navigate the data. The manually curated reference regulons are accessible via *regulog collections* and *browse* web pages, or by using a *keyword search* tool. The central *regulog collections* page has entry points to the pages with regulog classifications of six different types: by taxonomic groups, by TFs, by TF families, by RNA families, by effectors, and by metabolic pathways, as described in the previous section. The *browse by regulogs* and *browse by genomes* pages contain the complete lists of all studied regulogs and genomes, and give an alternative way to access each individual regulog and genome page in the database. A *keyword search* tool located in the top right corner on any RegPrecise page allows a user to search for target genes and regulators using their locus tags or names, and to access the corresponding regulon pages for particular genomes. A case study of the HexR regulon below describes major ways to access data and web interfaces in RegPrecise 3.0.

**Figure 3:**
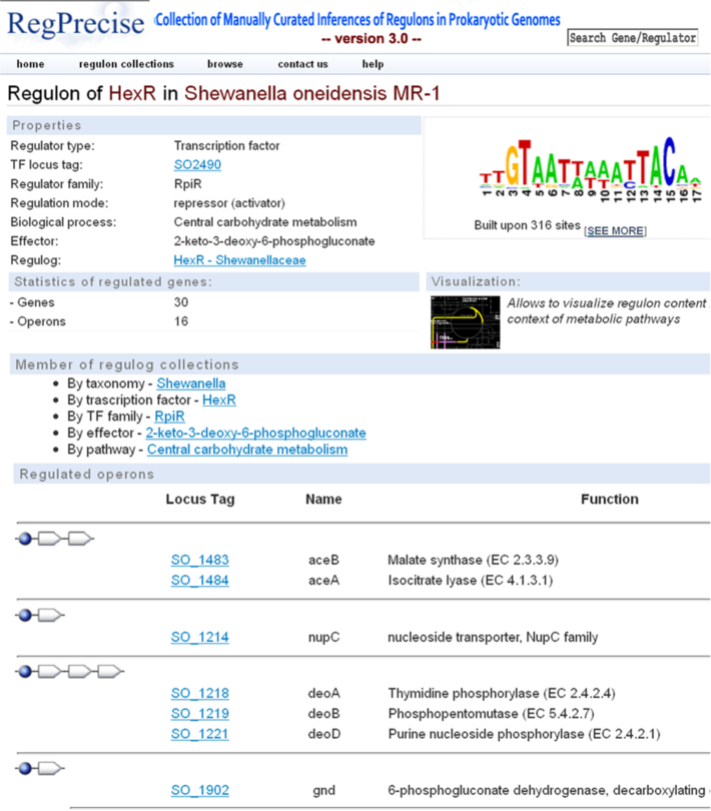
**The regulon page in RegPrecise.** The screenshot illustrates the details of the HexR regulon in *Shewanella oneidensis*.

**Figure 4:**
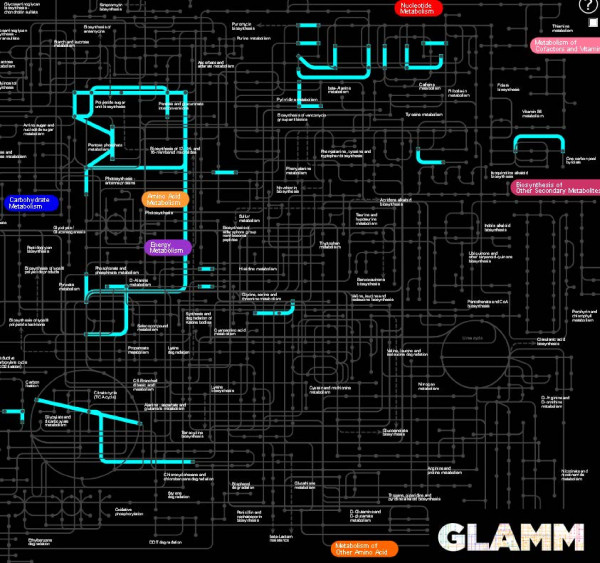
**A GLAMM representation of functional regulon content in RegPrecise.** The screenshot highlights metabolic pathways and reactions controlled by the HexR regulon in *Shewanella oneidensis*.

The *regulon* page (as illustrated by the HexR regulon in *Shewanella oneidensis* in Figure 3) gives a brief summary for a regulator (TF or RNA type, locus tag, family, regulation mode, regulated biological process, effector) and a complete list of predicted target genes/operons with their locus tags, names and functions. In addition, the regulon page contains cross-links to the parent regulog and regulog collections pages, the TFBS motif page, and the visualization page. The latter page utilizes the Genome-Linked Application for Metabolic Maps (GLAMM) interface and presents the functional overview of a regulon by visualizing its predicted members in the context of metabolic networks [41]. For instance, an image of the HexR regulon in *Shewanella* shows that it contains genes involved in the energy, carbohydrate and nucleotide metabolisms (Figure 4).

**Figure 5:**
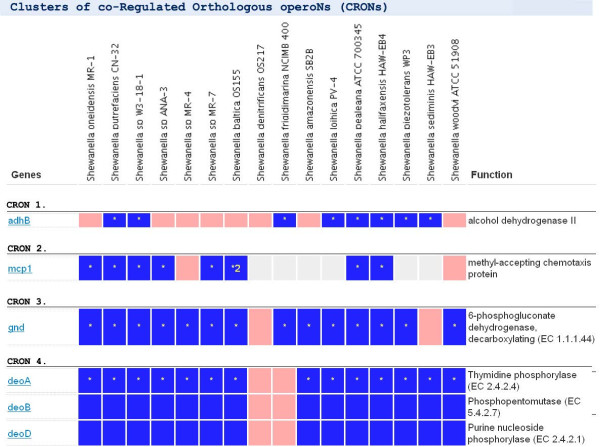
**The regulog page in RegPrecise.** The screenshot illustrates CRONs constituting the HexR regulog in the *Shewanella* taxonomic group.

The *regulog* page allows one to analyze the evolutionary conservation of gene regulation by orthologous regulators in a set of closely related genomes. A comparative table of all CRONs shows a phylogenetic profile of gene regulation by a regulator across the genomes (as illustrated by the HexR regulog in *Shewanellaceae* in Figure 5). The table of CRONs *allows one to identify* a core part of the regulog populated by genes with broadly conserved regulatory sites and a variable part of the regulog containing genes with non-conserved sites. The regulog page also has a link to the GLAMM [41] visualization of metabolic content of the entire regulog. For each TF-operated regulog, the TFBS motif logo has a link to the *profile* page containing detailed information about associated TFBSs (site sequence, score and position relative the gene start).

**Figure 6:**
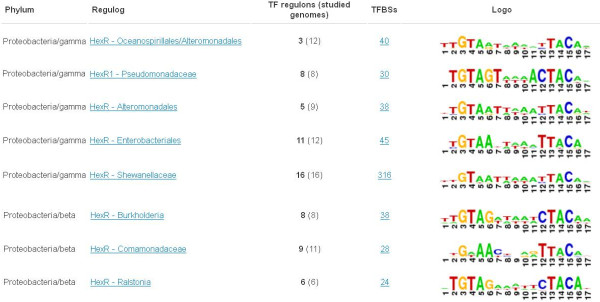
**The TF collection page in RegPrecise.** The screenshot illustrates the collection of HexR regulogs in various taxonomic groups of Proteobacteria.

The *collections* pages provide access, unique representation, description and summary statistics for regulogs grouped by several properties:

*Collections of TFs and TF families* (see HexR regulog collection in Figure 6) for each regulog contain unique alignments of TFBSs motif logos, which allow for evolutionary analysis of homologous TFs and their binding motifs.

*Collections of RNA families* represent and classify all RNA-operated regulons.

*Collections of effectors* facilitate the analysis of different regulators that respond to the same effector.

*Collections of pathways* identify different regulatory mechanisms for transcriptional control of the same metabolic pathway.

**Figure 7:**
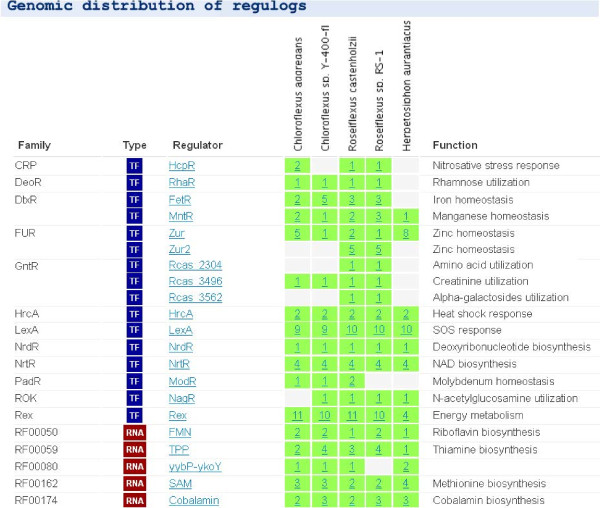
**The taxonomy collection page in RegPrecise.** The screenshot illustrates the collection of regulogs in the Chloroflexi taxonomic group.

*Collections based on taxonomy* give an overview for distribution of all reconstructed TF and RNA regulogs arranged by regulog type and family attributes in all analyzed genomes from the same taxonomic group. A taxonomy collection page (see the *Chloroflexi* regulog collection in Figure 7) highlights universally conserved and narrowly distributed regulogs, and provides cross-links to both individual regulog and genome pages.

**Figure 8:**
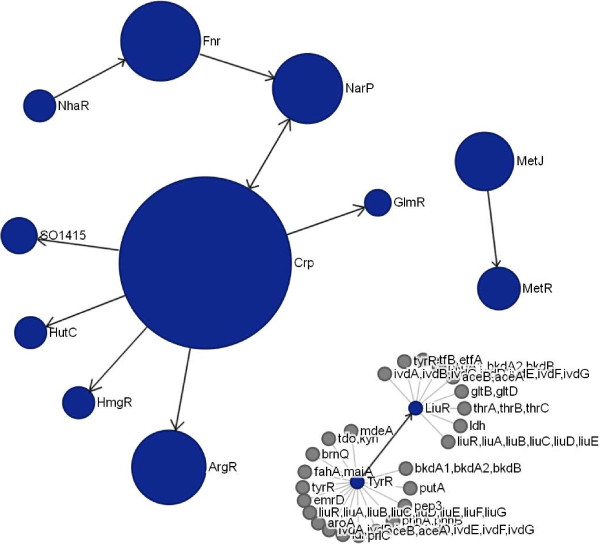
**The regulatory cascades on the genome page in RegPrecise.** The screenshot illustrates the regulatory cascades in the reconstructed regulatory network of *Shewanella oneidensis*.

**Figure 9:**
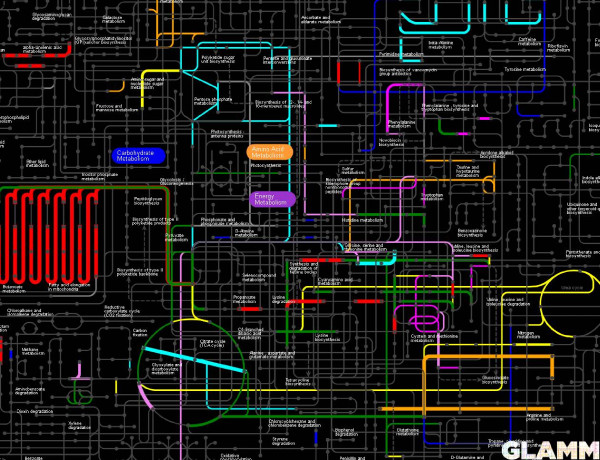
**The regulated metabolic pathways on the genome page in RegPrecise.** The screenshot illustrates the GLAMM representation of metabolic pathways controlled by all reconstructed regulons in *Shewanella oneidensis*.

A *genome* page summarizes information on all reconstructed TF and RNA regulons in a given genome, gives access to a functional overview and visualization of a genome-wide regulatory network. The reconstructed genome-centric TRNs are visualized via an interactive JavaScript widget with scale-up and scale-down functions. Two types of a cross talk between regulons from highly interconnected TRNs are shown as respective page tabs: (i) regulatory cascades (as illustrated for the *Shewanella oneidensis* TRN in Figure 8), and (ii) co-regulations of genes by multiple regulators. Finally, the functional content of an entire TRN in a given genomes is visualized via the GLAMM applet (as illustrated for the *Shewanella oneidensis* TRN in Figure 9).

The regulon, regulog and TFBS profile pages are linked to relevant datasets of co-regulated genes (as a tab-delimited text) or regulatory sites (as a fasta-formatted text) for export. In addition, the RegPrecise web services interface provides programmatic access to all regulatory interactions and regulon data in the database [22]. All locus tags for TFs and target genes in RegPrecise 3.0 have cross links to web pages in the MicrobesOnline genomic database [39].

We are planning to incorporate supportive experimental evidences for reconstructed regulons and effectors using information from literature and other databases on microbial regulation including RegTransBase [25], RegulonDB [26], DBTBS [27], and CoryneRegNet [24]. We are also planning to develop new graphic modules allowing the RegPredict-style representation of CRONs and species trees on the regulog page. The datasets of reference regulons will be expanded by novel collections for more than 20 taxonomic groups from both Bacteria and Archaea domains.

## Conclusions

The RegPrecise 3.0 is a significantly updated and enhanced version of an open-access database that contains reference collections of curated microbial regulons operated by TFs and RNA and inferred by the comparative genomics. The reference collections of TF regulons from 161 genomes were conservatively propagated to near 500 new genomes. The draft propagated regulons constitute a separate section in the database. RegPrecise provides a unique user-friendly representation of regulatory interactions with multiple interfaces to give access to multiple features of the inferred regulog collections at several hierarchical levels. Accumulated data on the regulatory interactions in diverse bacterial species will be useful for a broad scientific community. In particularly, these data can provide a basis for: 1) planning future experiments for validation of novel regulatory mechanisms inferred by comparative genomics; 2) analyzing evolution of microbial regulatory networks; 3) building predictive biological models combining regulatory and metabolic networks.

## Availability and requirements

RegPrecise 3.0 is freely available at http://regprecise.lbl.gov.

## Abbreviations

TF: Transcription factor; TFBS: Transcription factor-binding site; TRN: Transcriptional regulatory network; PWM: Position weight matrix; CRON: Cluster of co-regulated orthologous operons.

## Competing interests

The author declares that they have no competing interests.

## Authors’ contributions

PSN and DARo directed the whole research, designed the database, and wrote the manuscript. PSN developed the database. AEK and GYK developed controlled vocabularies of effectors and pathways. MDK identified riboswitches. AEK, DARo, DARa, and SAL performed comparative genomic analysis to infer transcriptional regulons. DARo curated regulon reconstructions. WR implemented the GLAMM module. RAS implemented regulatory networks visualizations. ID and APA contributed to the design of the study, discussed results and critically revised the manuscript. All the authors have read and approved the final version of the manuscript.

## Supplementary Material

Additional file 1: **Domain architectures and protein family classification of TFs in RegPrecise.** Each TF family has an assigned domain rule containing known domains from Pfam, COG and Superfamily databases. Text descriptions of TF families include literature references in PubMed. Click here for file
